# Supplementary material for: Parental state dynamically reshapes auditory processing of offspring vocalizations in zebra finches
Source: J Neuroendocrinol. 2026 Sep 10;38(9):e70266. doi: 10.1111/jne.70266 (PMC13559750; doi:10.1111/jne.70266)
Supplement: Supplementary file 1 [file JNE-38-e70266-s001.pdf]

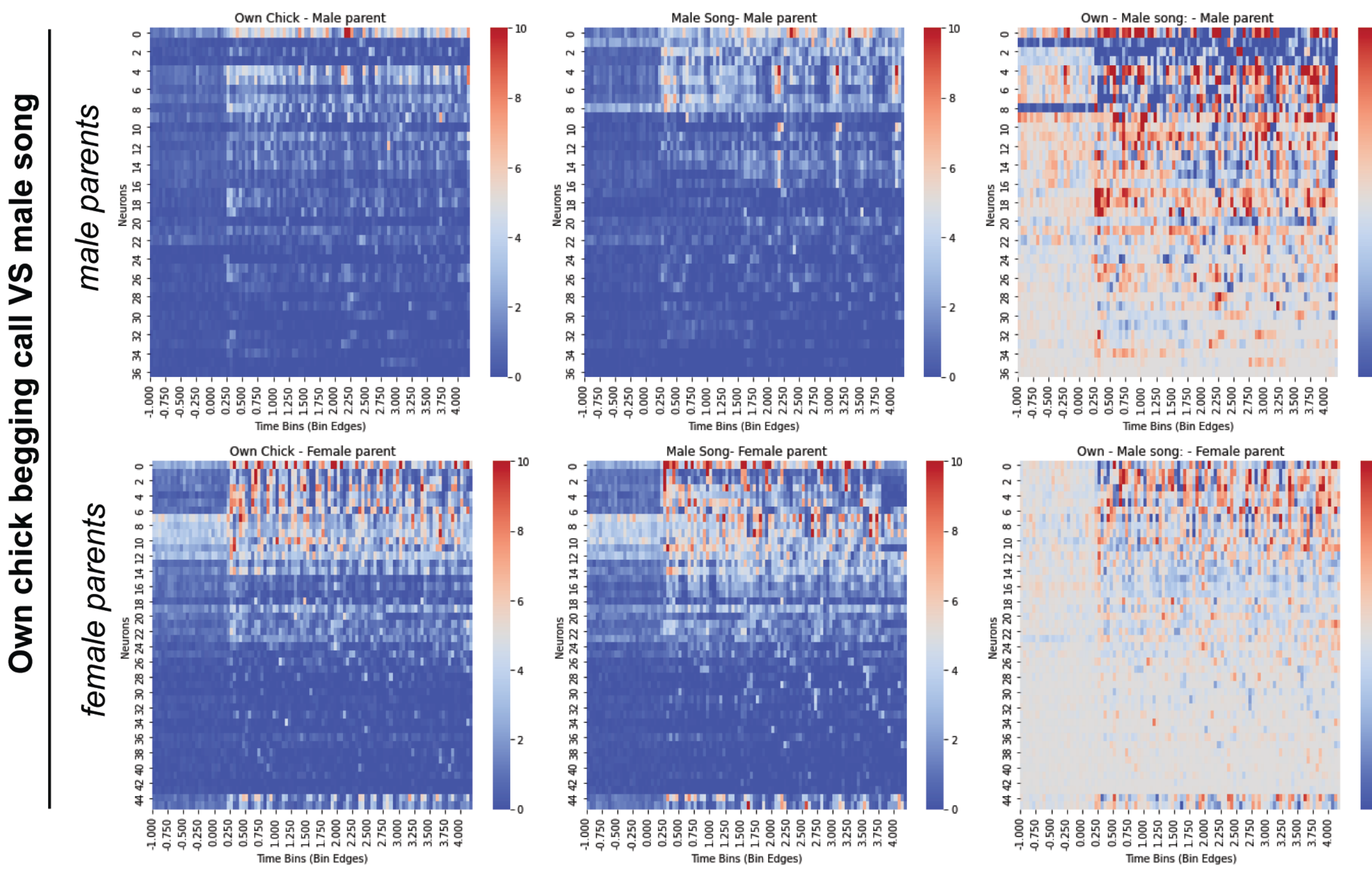
Own chick begging call VS male song
male parents
female parents
Own Chick - Male parent
Male Song- Male parent
Own - Male song: - Male parent
Own Chick - Female parent
Male Song- Female parent
Own - Male song: - Female parent
Neurons
Time Bins (Bin Edges)
